# Exploring Galectin‑3 as an Emerging Frontier in Biomarker Research for Clinical Trials Approaching Cardiovascular Diseases

**DOI:** 10.1021/acsptsci.5c00671

**Published:** 2026-03-11

**Authors:** Luís Perpétuo

**Affiliations:** 1 iBiMED, Department of Medical Sciences, 451098 University of Aveiro, Campus Universitário de Santiago, Aveiro 3810-193, Portugal; 2 Cardiovascular R&D Centre − RISE-Health, Department of Surgery and Physiology, Faculty of Medicine of the University of Porto, Alameda Professor Hernâni Monteiro, Porto 4200-319, Portugal

**Keywords:** clinical trials, galectin-3, biomarkers, heart failure, myocardial infarction

## Abstract

Research has consistently highlighted galectin-3 (Gal-3) as a leading biomarker candidate for cardiovascular and cerebrovascular disease. Within studies focused on atherosclerosis-related cardiovascular disease, Gal-3 has played a multifaceted role, from monitoring disease progression to predicting outcomes. This reanalysis highlights the association of Gal-3 with various clinical factors, pathophysiological processes, other biomarkers, and critical outcomes. To maximize the potential of Gal-3 in future research, a uniform methodology that ensures consistent and easily comparable results is essential. Furthermore, our results highlight the synergy between elevated Gal-3 levels and established diagnostic measures of heart failure (HF). In this review, we present a reanalysis that provides a seminal synthesis of clinical trials using Gal-3 as a measure or outcome measure and aims to both capture current understanding and pave the way for its increased clinical application.

## Graphical abstract

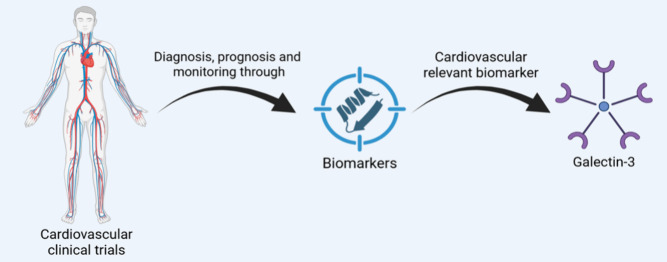

## Novelty and Significance

Current Understanding:

1. Galectin-3 (Gal-3) is involved in numerous biological functions, from cell proliferation to pathophysiological processes such as fibrosis and atherosclerosis.
2. Given its role in inflammation and atherosclerotic events, Gal-3 has been investigated for its biomarker potential in cardiovascular disease.

**New Insights from This Article:**

1. Associations of galectin-3 span a broad spectrum, from biomarkers and clinical factors to disease outcomes, increasing its importance as a biomarker for cardiovascular disease.
2. Elevated Gal-3 levels show significant concordance with left ventricle ejection fraction (LVEF) and NT-proBNP, as well as frailty-associated factors such as age, diabetes, estimated glomerular filtration rate (eGFR), and administration of diuretic medication, highlighting its biomarker potential specifically in heart failure.
3. Clinical trials investigating diseases such as myocardial infarction, stroke, and heart failure could benefit from comprehensive and/or standardized patient histories and regular measurements of biomarkers, especially Gal-3, accompanied by rigorous monitoring using tools such as echocardiography, magnetic resonance imaging (MRI), and angiograms.

Cardiovascular diseases (CVDs) are responsible for almost a third of deaths yearly and are expected to reach nearly 24 million annually by 2030.^1^ CVDs share several risk factors, such as high blood pressure, high blood cholesterol, smoking, diabetes, age, and family history.^2^ Most CVDs are clinically silent in their early stages and are often detected in later stages. Aiming at an earlier diagnosis, biomarkers to diagnose and predict CVDs have been on the rise of interest. Currently, suggested biomarkers for atherosclerotic diseases include von Willebrand factor (vWF), IL-6, high-sensitivity C-reactive protein (hs-CRP), C-reactive protein (CRP), myeloperoxidase (MPO), matrix metallopeptidase 2 (MMP2), matrix metallopeptidase 9 (MMP9), and metalloproteinase inhibitor 1 (TIMP1).^3,4^ NT-proBNP, BNP, high-sensitivity cardiac troponin (hs-cTn), ST2, and GDF-15 have been pointed out as biomarkers for myocardial infarction (MI).^5–7^ TNF-α, VCAM 1, ICAM 1, and GFAP have been associated with ischemic stroke.^4^ Additionally, galectin-3 (Gal-3) has become a hot topic for its potential as a CVD biomarker.^8^

Gal-3 is a β-galactosidase-binding lectin that can be found in many compartments, such as the cytoplasm, nucleus, cell surface, and even in the extracellular environment,^8^ which explains its myriad of functions (Figure 1). Gal-3 is involved in numerous biological processes such as cell proliferation, inflammation, fibrosis, and apoptosis regulation.^9–11^ Moreover, Gal-3 is involved in cell–cell and cell–matrix interactions, adhesion, and immunity processes.^12,13^ In the cytoplasm, Gal-3 is involved in inducing proliferation and in antiapoptotic regulation;^14,15^ in the nucleus, it is involved in the regulation of gene transcription;^15^ in the cell surface, it has an important role in lattice assembly, which is, for instance, responsible for selecting, activating, and arresting T-cells;^16^ and in the extracellular environment, through binding to different cell surfaces and extracellular matrix glycans, Gal-3 induces cell adhesion and migration, regulates growth, and displays an overall pro-apoptotic effect (Figure 1).^17,18^ This pleiotropy confers Gal-3 relevant roles in many pathophysiological phenomena, such as fibrosis or atherosclerosis, and in many conditions, such as heart failure (HF).^12^

**Figure 1:**
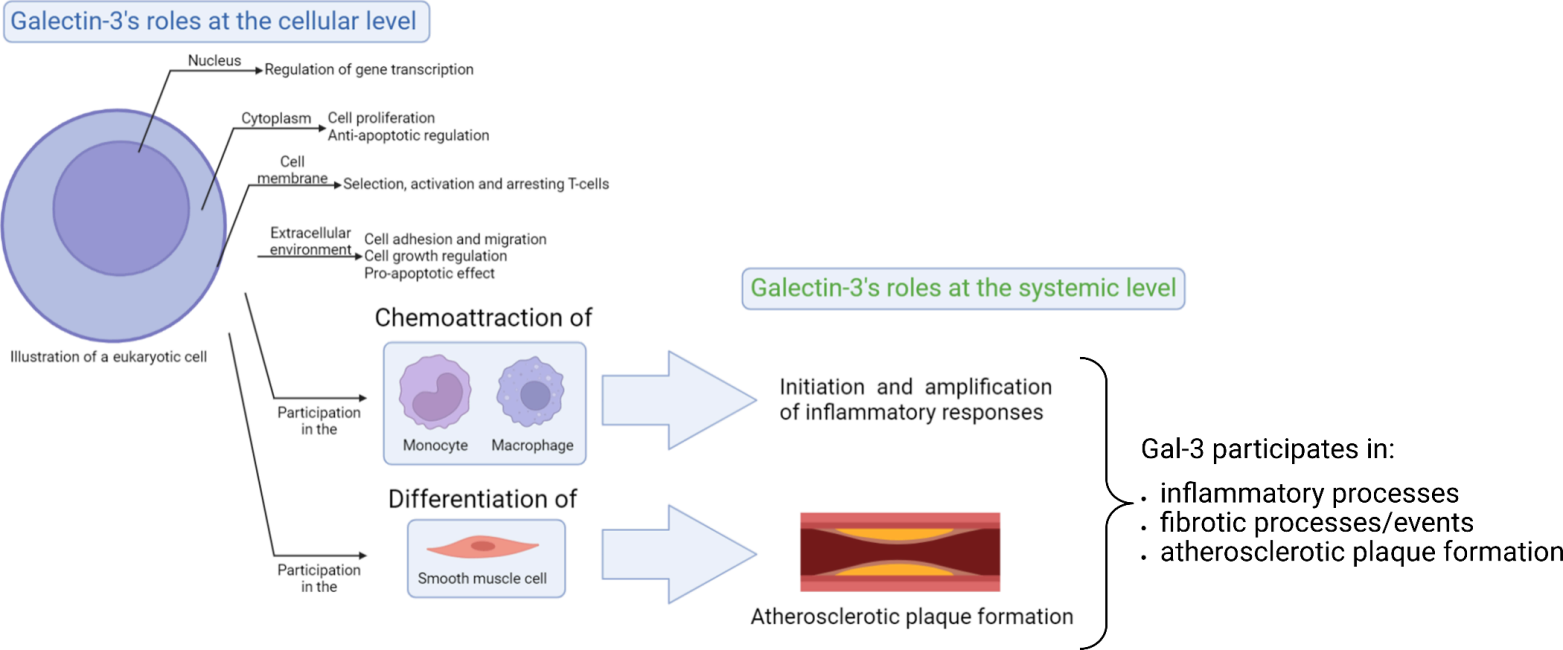
Cellular and systemic involvement of Gal-3 in atherosclerotic cardiovascular disease.

Gal-3, as an inflammatory protein, is involved in the chemoattraction of monocytes and macrophages^19^ and contributes to the initiation and amplification of inflammatory responses.^9^ In particular, Gal-3 has been emerging as a potential inflammatory mediator in atherosclerosis.^20^ Gal-3 was found to regulate chronic inflammation by promoting the differentiation of vascular smooth muscle cells (VSMCs) into osteoblast-like cells, which promote atherosclerotic plaque calcification (Figure 1).^21,22^ Moreover, a study found that overexpressing Gal-3 in VSMCs induced an increase in collagen type I synthesis, suggesting that it might be involved in vascular wall remodeling.^23^ It is also suggested that Gal-3 partakes in atherosclerosis plaque exacerbation by promoting endocytosis of oxidized LDL (ox-LDL) by foam cells.^20^

Being involved in many inflammatory processes and in atherosclerotic plaque formation, naturally, many studies have explored the potential of Gal-3 as a diagnostic and/or prognostic biomarker for heart diseases.^24–28^ Some clinical trials have already tried to validate Gal-3 as a CVD biomarker, and some have already used this protein as an established biomarker for disease monitoring or prognosis, as well as a monitoring tool for CVD therapy outcome.^29,30,39,40,31–38^ Gal-3 can be found in blood and urine, providing media for a non- or minimally invasive assessment of this protein.^9^ Here, we propose to reanalyze clinical trials to highlight Gal-3′s associations with CVD biomarkers, clinical factors, and outcomes, emphasizing its potential as a diagnostic and prognostic tool. It also underscores the need for standardized methodologies and synergy with existing measures in heart failure and atherosclerosis-related conditions.

## Methods

In most clinical trials, Gal-3 was measured in serum^29,31,44–47,33,36–38,40–43^ or plasma,^32,34,54–63,39,64,47–53^ but also in myocardial tissue.^39^ In the majority of the studies Gal-3 is measured by enzyme-linked immunosorbent assay (ELISA) or by the more advanced chemiluminescent microparticle immunoassay (CMIA).^29,45,54,61^ Blood samples were often collected 24–96 h after hospital admission or right before discharge. Plasma and serum were separated soon after (up to 1–2 h) by centrifugation at 3500 rpm (1,500*g* approximately) for 15 min at room temperature. Then, when necessary, samples were stored at −80 °C until analyzed. Depending on the study design, Gal-3 was measured only once (baseline, 0 time point),^31,42,43,45,49,51–55,58^ or additionally 2–6-months after the baseline^29,31,54,58^ and even 8–18 months after.^31,42^ In one case, Gal-3 was measured at the baseline and 72 h after receiving treatment.^59^ In one experiment, myocardial tissue was obtained via biopsy from individuals who suffered accidental death.^39^

This reanalysis was performed using a query on “Clinical trials.gov” using the keywords “cardiovascular disease” and “galectin-3”. As of October 20, 2023, this query returned 74 results. Assessing these 74 articles, 31 did not meet the criteria selected for this review – original research in a human cohort, more specifically, clinical trials. Out of the remaining 43 articles, 29 ( ^29,31,41–43,47–50,53–55,32,56–64,33,34,36–40^ ) had relevant Gal-3 associations with cardiovascular diseases’ outcomes, comorbidities, clinical factors, and underlying pathophysiological processes, therapies used in these diseases; and other biomarkers assessed in these studies, and thus were included in this reanalysis (Figure 2).

**Figure 2:**
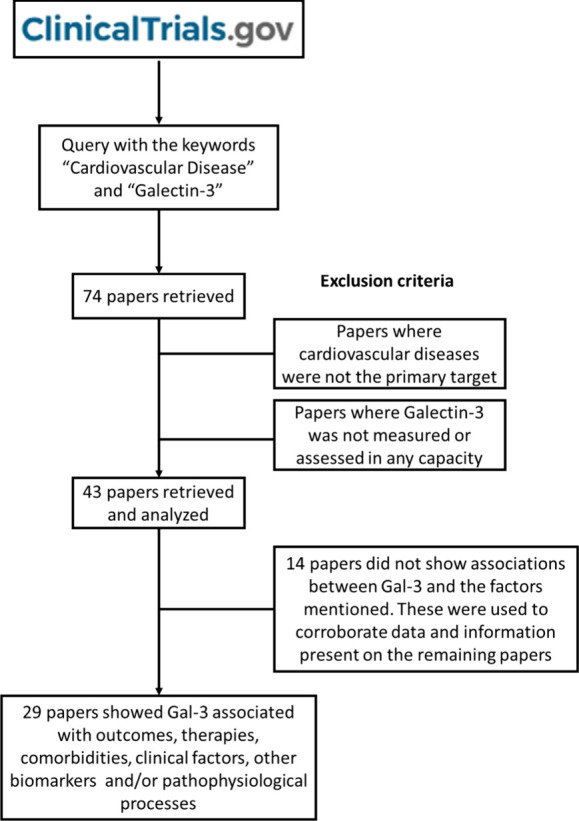
Study inclusion and exclusion criteria: a query on “ClinicalTrials.gov” with the keywords “cardiovascular disease” and “galectin-3”, returned 74 results, of which 43 were relevant for the review at hand. Twenty-nine of these results had relevant/important associations between Gal-3 and CVDs, pathophysiological processes, therapies/medications, biomarkers, comorbidities, and clinical factors.

## Results and Discussion

Gal-3 was assessed in clinical trials on stroke, myocardial infarction (MI), aortic valve stenosis (AVS), and HF. The previously mentioned associations regarding Gal-3 (associations with cardiovascular diseases’ outcomes, comorbidities, clinical factors, and underlying pathophysiological processes; therapies used in these diseases; and other biomarkers assessed in these studies) are summarized and highlighted in Table 1.^57^

**Table 1:** Associations Found Regarding Gal-3 in the 29 Analyzed Clinical Trials That Passed the Exclusion Criteria (Figure 2)

category of association	subcategory of association	associated factor
diseases	cardiovascular system	aortic valve stenosis (AVS)^31^
		atrial fibrillation (AF)^32,61^
		hypertension^39,61^
comorbidities/clinical factors	cardiovascular system	heart rate^49,58^
		lower blood pressure^49,56^
		previous history of coronary artery bypass graft (CABG)^58^
	kidney system	estimated glomerular filtration rate (eGFR) (negatively)^39,49,51,58^
		worse renal function^51,55,56,64^
	pancreas system	diabetes^48,56^
	age and lifestyle	age (younger),^29^ age (older)^31,39,47–49,55–61,64^
		nonsmoker^47^
		nondrinker^47^
pathophysiological processes	cardiac remodeling	concentric hypertrophy^31^
		relative wall thickness^31,56^
		left ventricular myocardial index^39^
		fibrosis^29,31,46^
	diastolic disfunction	left atrial diameter^32,61^
		E/E’ ^36,61^
		E’^36^ (negatively)
		left atrial volume index (LAVI)^36^
	systolic disfunction	left ventricular diastolic dysfunction (LVDD)^36,50^
		lower left ventricle ejection fraction (LVEF)^48,49,51,58,60^
	arrhythmias	atrial fibrillation (AF)^49,55^
		microvolt T-wave alternans (MTWA)^39^
biomarkers		NT-proBNP/BNP^33,45,48,49,51,55,56,58,61,64^
		hs-CRP^29,49,57,58^
		aldosterone^48,49,58^
		collagen type-1 C-terminal telopeptide (CITP)^33,45,56^
		P3NP^48,56^
		hs-troponin^51,56^
		sST2 ^55^
		CK-MB^60^
		PICP^29^
		MMP1 ^33^
		MMP3 ^59^
		serum creatinine^59^
		TIMP1 ^59^
		IL8 ^59^
outcome risk assessment		HF risk stratification^34,44,57^
		myocardial infarction risk stratification (MI)^35^
		risk of vascular events 1 year after stroke^38,47^
outcomes	cardiovascular outcome	CV death^29,33,44,49,51^
		CV hospitalization^49^
		major acute cardiovascular events (MACE)^29^
	HF outcome	HF hospitalization^33,48,51,61^
		rehospitalization^34^
		worse prognosis^33,46,48,51,61^
		increased risk of CV events^64^
	myocardial infarction outcome	myocardial infarction infarct size 4 months after myocardial infarction prediction^60^
	stroke outcome	stroke recurrence^38,47^
		increased risk of death or major disability after stroke^40^
therapies		sacubitril/valsartan^46^
		mineralocorticoid receptor antagonists (MRA) therapy (patients with higher levels of Gal-3 benefited more from this therapy)^64^ and similar behavior for rosuvastatin^58^
		canrenone^48^
		simvastatin^50^
		valsartan was more effective in patients with low Gal-3 ^51^
		CD34+ therapy^52^
		*N*-acetylcysteine (NAC)^53^
		eplerenone^54^

### Clinical Relevance of Gal-3

Taking into consideration the number of associations shown in Table 1, some clinical trials showed more significant associations between Gal-3 levels and outcome/disease/comorbidity/biomarkers/therapy than did others. We have collected the data from some of the clinical trials that showed the most associations with Gal-3 levels in order to establish a profile of what insight this protein level can provide, regarding disease risk assessment and diagnosis (in particular HF and MI). Table 2 provides information regarding patients with elevated Gal-3 levels. For each study, statistically significant differences between the cohort with elevated Gal-3 levels and the other cohorts were indicated by an asterisk. The data indicate that age, diabetes, left ventricle ejection fraction (LVEF) (%), estimated glomerular filtration rate (eGFR), administration of diuretic medicine, and NT-proBNP levels are correlated with elevated levels of Gal-3. Certainly, the correlation between Gal-3 levels and LVEF (a strong indicator for the diagnosis of HF) and NT-proBNP (a circulating biomarker that has been used as a tool for the diagnosis of HF) shows potential of Gal-3 to also be associated with the pathophysiology of this condition and therefore being a biomarker for HF as well.

**Table 2:** Clinical Data, Comorbidities, and Medical History Collected in the Clinical Trials That Showed the Highest Number of Associations with Gal-3 Levels (Table 1) (*Statistically Significant Data for Each Individual Clinical Trial)^a^

	MI	HF	HF	HF	HF	HF	Reference values
paper reference	47	48	49	56	58	61	
*N* of associations with gal-3	7	9	10	9	9	6	N.A.
*N*	1069 (total = 2970)	205 (total = 413)	488 (total = 1462)	69 (total = 208)	728 (total = 1462)	207 (total = 415)	N.A.
galectin-3 (ng/mL)	>8.65	13.3 [12.0–15.8]	>21.6	21.0 [18.1–24.7]	>19.0	>12.1	∼5–15 (assay-dependent)
age (years)	64.2 ± 10.89*	65.0 ± 8.3*	74 ± 7*	71* ^65–79^	73 ± 7*	68 ± 8*	N.A.
female (%)	45.89*	16.6	29*	46	27*		N.A.
BMI (kg/m2)	24.81 ± 3.18*	27.0 ± 3.8	27.3 ± 5	32.8 [28.1–39.6]	27.2 ± 4.98	29.3 ± 3.6	18.5–24.9
total cholesterol (mmol/L)	5.3 ± 1.3*		5.24 ± 1.17		5.23 ± 1.13		<5.2
LDL cholesterol (mmol/L)	3.07 ± 1.05*		3.62 ± 1.02		3.63 ± 1.04		<3.0 (optimal <2.6)
SBP (mmHg)	166.7 ± 17.19	128.8 ± 17.9	129 ± 17*		129 ± 17*	133.8 ± 18.8	90–120
BDP (mmHg)	96.26 ± 11.37	78.2 ± 8.5	76 ± 10*		79 ± 9*	77.5 ± 11.6	60–80
HR (beats/min)		67.9 ± 11.5	72 ± 11*		72 ± 11*		60–100
current smoker (%)	29.81*		11		12		0
hypertension (%)	80.88*	49.3	68	91	69	94.7*	0
diabetes (%)	26.1*	26.3*	28	57*	28*	19.8	0
myocardial infarction (%)		45.9	67		65*	17.9	0
CABG or PCI (%)			25*		23*	8.7	0
prior hospitalization for HF (%)		56.1*		45		33.8	0
COPD (%)		14.2*		19		3.9	0
peripheral vascular disease (%)		5.4*				5.8*	0
LVEF (%)		38.5 ± 8.6*	30.9 ± 6.8*		31 ± 7*	67.8 ± 7.9	52–72 (men), 54–74 (women)
left atrial diameter (cm)		4.3 ± 0.8*					≤4.0
aspirin (%)	34.72					18.8*	0
diuretics (%)		78.1*	92*	93*	89*	63.8*	0
B-blockers (%)	54.26*	78.5	73	80	74	79.2*	0
ACEi or ARBs (%)		100*	81/43*	75	81	81.2	0
digitalis (%)		25.4	32*		31		0
serum creatinine (mg/dL)		1.11 ± 0.28*		1.3 [1.0–1.7]*			0.6–1.3
PIIINP (ug/L)		5.8 [4.4–7.4]*		8.3 [6.7–11.5]*			∼2.3–6.4
NT-proBNP (pg/mL)			2089 [820–3983]*	1187 [397–2205]*	213.2 [88.7–444.2]*	192 [93–377]*	<125 (<75 yrs), < 450 (≥75 yrs)
BNP (ng/L)		112 [52–222]*					<100
hsCRP (mg/L)			4.7 [2.2–9.8]*	3.8 [1.7–8.5]	4.6 [2.1–8.9]*		<3
triglycerides (mmol/L)	1.89 ± 4.53*		2.08 ± 1.51		2.07 ± 1.41		<1.7
eGFR (mL/min per 1.73 m2)			49 ± 13*	51* ^35–68^	52.0 ± 13.3*	72.5 ± 18.2*	≥90
apoB/apoA-1 ratio			0.91 ± 0.27*		0.9 ± 0.26		<0.7
uric acid (mg/dL)				8.0 [6.4–9.6]*			3.5–7.2 (men), 2.6–6.0 (women)

a Reference values correspond to typical adult clinical laboratory ranges in the general population and are provided for contextual comparison only. They do not represent disease-specific targets or eligibility criteria and may vary according to age, sex, assay, and clinical setting. Abbreviations: SBP – systolic blood pressure, DBP – diastolic blood pressure, CABG – coronary artery bypass graft, PCI – percutaneous coronary intervention, HR – heart rate, COPD – chronic obstructive pulmonary disease, LVEF – left ventricle ejection fraction, eGFR – estimated glomerular filtration rate.

A study found that patients with myocardial infarction that presented Gal-3 levels above 8.65 ng/mL, versus a cohort with lower levels, had significant differences regarding age, sex, body mass index (BMI), total cholesterol, LDL cholesterol, triglycerides, smoking status, occurrence of hypertension, diabetes, and taking medications such as beta blockers, angiotensin-converting enzyme inhibitors (ACEi), or angiotensin receptor blockers (ARBs).^47^

For patients with heart failure, those exhibiting elevated levels of Gal-3 (13.3 ng/mL,^48^ >21.6 ng/mL,^49^ 21.0 ng/mL,^56^ >19.0 ng/mL,^58^ and >12.1 ng/mL^61^) consistently presented several statistically significant differences when compared with cohorts with lower Gal-3 levels. These findings were reported across heterogeneous HF populations, including survivors of acute myocardial infarction with subsequent HF development,^48^ patients with chronic HF with reduced ejection fraction (HFrEF),^49,56,58^ and patients hospitalized for acutely decompensated HF.^61^ In these studies, Gal-3 stratification was performed using either predefined cutoffs or median-based cohort division, and associations were evaluated using univariate and multivariate analyses adjusted for relevant clinical covariates.

Across these cohorts, elevated Gal-3 was associated with older age^48,49,56,58,61^ and, in some studies, with sex differences.^48,58^ Patients with higher Gal-3 also exhibited worse hemodynamic profiles, including higher heart rate and lower systolic and diastolic blood pressure,^49,58^ as well as a higher prevalence of hypertension^61^ and diabetes mellitus.^48,56,58^ A more advanced cardiovascular disease burden was evident, with higher frequencies of prior myocardial infarction,^65^ previous coronary revascularization procedures (CABG or PCI),^49,58^ and prior hospitalizations for HF.^48^ Additionally, elevated Gal-3 was associated with extracardiac comorbidities, including chronic obstructive pulmonary disease (COPD)^48^ and peripheral artery disease (PAD).^48,61^

From a cardiac structural and functional standpoint, higher Gal-3 levels were consistently linked to worse systolic and diastolic parameters, including lower left ventricular ejection fraction (LVEF)^48,49,58,61^ and increased left atrial diameter,^48^ reinforcing its association with adverse cardiac remodeling. Regarding pharmacological profiles, patients with elevated Gal-3 were more frequently treated with aspirin,^61^ diuretics,^48,49,56,58,61^ beta-blockers,^61^ ACE inhibitors or angiotensin receptor blockers,^48,49^ and digitalis,^49^ reflecting more advanced disease severity and symptom burden.

Biochemically, elevated Gal-3 was associated with markers of renal dysfunction and systemic inflammation, including higher serum creatinine and PIIINP,^49,56^ reduced estimated glomerular filtration rate (eGFR),^49,56,58,61^ increased NT-proBNP^49,56,58,61^ and BNP levels,^48^ elevated hs-CRP,^49,58^ higher uric acid,^56^ and altered lipid-related indices such as the ApoB/ApoA-1 ratio.^49^ Collectively, these findings indicate that elevated Gal-3 identifies HF patients with a more adverse clinical, biochemical, and functional profile across different HF phenotypes and clinical settings.

We focused on the parameters that showed more statistically significant differences among the analyzed studies: age, diabetes, LVEF, diuretics, NT-proBNP, and eGFR. Because the referenced studies have different cohort sizes and the Gal-3 values are differently categorized (some studies mention median with interquartile range 1 and 3, while other studies mention the minimum value of Gal-3 measured in that cohort), we will reference the lowest and highest value of Gal-3 and the corresponding lowest and highest value of the comorbidity (e.g., age) to provide a range of values.

For HF, the lowest value of Gal-3 considered for the cohorts with worse prognosis was 12.1 ng/mL, and the highest was at least 21.6 ng/mL. Considering the comorbidities previously mentioned, for this range of levels of Gal-3, we find ages ranging from 65.0 ± 8.3 to 74 ± 7 years, a frequency of diabetes ranging between 26.3 and 57%, a LVEF ranging from 30.9 ± 6.9 to 38.5 ± 8.6%, a frequency of taking diuretic medication and eGFR ranging between 63.8 and 83%, and 49 ± 13 mL/min per 1.73 m^2^ and 72.5 ± 18.2 mL/min per 1.73 m^2^, respectively. Additionally, NT-proBNP was found in the range of 192 [93–337] to 2089 [820–3983] pg/mL. To note, the ranges mentioned only referenced values that were statistically significant between each cohort and other cohorts (less impacted by the disease/controls).

Age is a factor that contributes to fragility, a geriatric syndrome characterized by a multidimensional and cumulative decline in many organs and systems. It is expected that Gal-3, a pro-inflammatory protein, has a level increment with aging and is associated with other comorbidities.^65^ However, Komici et al. found that Gal-3 is independently associated with frailty in elderly patients with systolic HF.^66^ Similarly, the association between diabetes, eGFR, and the administration of diuretics is also associated with degenerative pathophysiology where the pancreas and the kidneys start to lose function, respectively. With diabetes, Gal-3 promotes the inflammation of pancreatic islet β cells and insulin target organs, which leads to pancreatic β cell failure and insulin resistance.^67^ Moreover, Gal-3 levels are associated with renal function (negative correlation with eGFR and positive correlation with the administration of diuretics).^68^ Nonetheless, all these factors play a role in the severity of HF, and therefore, Gal-3 transpires the presence and severity of HF.

NT-proBNP is a biomarker strongly associated with mortality in patients with HF, among other cardiovascular diseases such as coronary artery disease.^69^ The statically significant association between this already validated biomarker for HF and Gal-3 shows a heightened potential for Gal-3 to be indeed directly associated with the severity of HF and the potential of this protein to be used as a biomarker and a clinical tool in the diagnosis of HF.

In summary, these clinical trials, although very distinct in their approach and methodology, show that galectin-3 can be a valid biomarker for cardiovascular conditions, in particular, for HF. Figure 3 shows a network of associations between HF and MI, the different values/ranges of Gal-3 levels, and the several comorbidities, biochemical factors, previous medical procedures/medical history, medication, and other biomarkers.

**Figure 3:**
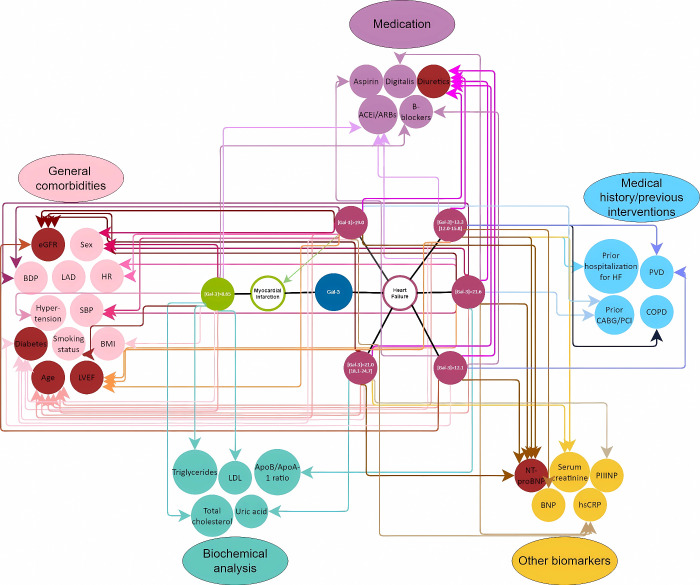
Network of associations between HF and MI, the different values/ranges of Gal-3 levels, and the several comorbidities (pink), biochemical factors (cyan), previous medical procedures/medical history (blue), medication (purple), and other biomarkers (yellow). Highlighted in red are the factors that were associated with Gal-3 levels more often (associations are meant as statistically differences between the cohort with worse prognosis and the cohorts with better prognosis/control cohort).

### Association of Gal-3 with Pathophysiological Processes

As mentioned previously, Gal-3 is involved in a multitude of biological processes. For instance, Gal-3 can activate fibroblasts, inducing collagen synthesis and decreasing collagen degradation.^70^ This may explain why Gal-3 is positively correlated with fibrosis.^39^ Zhou et al., 2015 demonstrated that in aortic valve stenosis, a higher degree of myocardial fibrosis is accompanied by a higher expression of Gal-3 in plasma and myocardial tissue.^39^ Intricately associated with fibrosis is the impairment of myocardial relaxation, which is a substrate for diastolic dysfunction. In this regard, plasma Gal-3 levels were found to be significantly correlated with early diastolic mitral annular velocity, early diastolic transmitral velocity to early diastolic mitral annular velocity, and left atrial volume index (LAVI).^34^ Also, higher levels of Gal-3 are associated with increased severity of diastolic dysfunction.^34^ Inflammation has been pointed as a link between Gal-3 and the development of left ventricular diastolic dysfunction (LVDD).^34^

Figure 3 provides an integrative, qualitative network representation of the associations identified across the analyzed clinical trials, focusing primarily on HF and MI. The network summarizes statistically significant associations between Gal-3 levels and clinical comorbidities, biochemical parameters, prior medical history, pharmacological therapies, and established cardiovascular biomarkers, as reported in the studies included in this reanalysis. Nodes represent individual variables, grouped by category (comorbidities, biomarkers, medication, and clinical history), while edges denote reported statistically significant differences between cohorts with higher versus lower Gal-3 levels. The network does not imply causality or directionality but rather reflects the frequency and consistency of reported associations across heterogeneous study designs and patient populations.

### Association of Gal-3 with Common Comorbidities

Galectin-3 levels are also associated with common CVD comorbidities. HF patients with higher Gal-3 levels were generally older, had lower body surface area, were more likely to have diabetes, and presented worse renal function.^62^ Patients with worse renal function are often medicated with diuretics, and elevated Gal-3 was found associated with diuretic medication intake as well as decreased estimated glomerular filtration rate (eGFR).^37,62^ Increased levels of Gal-3 were also found in patients with hyperglycemia, within 1 year after stroke.^36^ How hyperglycemia and Gal-3 are associated remains to be clarified, although it is known that patients with hyperglycemia are prone to elevated inflammatory cytokines in circulation.^71^ Furthermore, regarding myocardial fibrosis, the left ventricular myocardial index (LVMI) and Gal-3 were found higher in patients with hypertension, being associated with ECG-based microvolt T-wave alternans (MTWA). This association may be useful to assess the risk of cardiovascular events in hypertensive patients.^37^ A study on HFpEF reported an association between baseline hypertension and baseline Gal-3 levels, with more patients showing hypertension when Gal-3 levels were above 12.1 ng/mL (median level of Gal-3).^61^ However, other factors such as age, sex, and BMI among others that are correlated with hypertension and Gal-3 levels may influence the association between hypertension and Gal-3. Further studies are needed to validate this association and clarify the underlying mechanism that links Gal-3 with hypertension.

### Association of Gal-3 with Usual CVD Biomarkers

The association of Gal-3 with other fibrotic biomarkers such as osteopontin and gremlin-1 was found useful in the stratification of patients based on the risk for developing acute HF.^47^ Gal-3 has also been associated with other biomarkers to diagnose and prognosticate different CVDs. High levels of NT-proBNP, endothelin-1, hs-cTnI, and fibrosis biomarkers (P3NP and CTX) have been associated with higher levels of Gal-3. Aldosterone, however, has not been associated with higher levels of Gal-3,^62^ despite its role in myocardial and vascular fibrosis.^72^ High levels of aldosterone have been previously associated with worse HF prognosis.^30^ Moreover, the correlation between Gal-3 and NT-proBNP baseline levels suggested that left ventricular dysfunction involves both a myocardial and extracellular cardiac matrix remodeling.^31,48^ Indeed, Gal-3 elevated levels have been correlated with NT-proBNP in survivors of acute MI.^48^ Some of these associations are highlighted in Figure 4.

**Figure 4:**
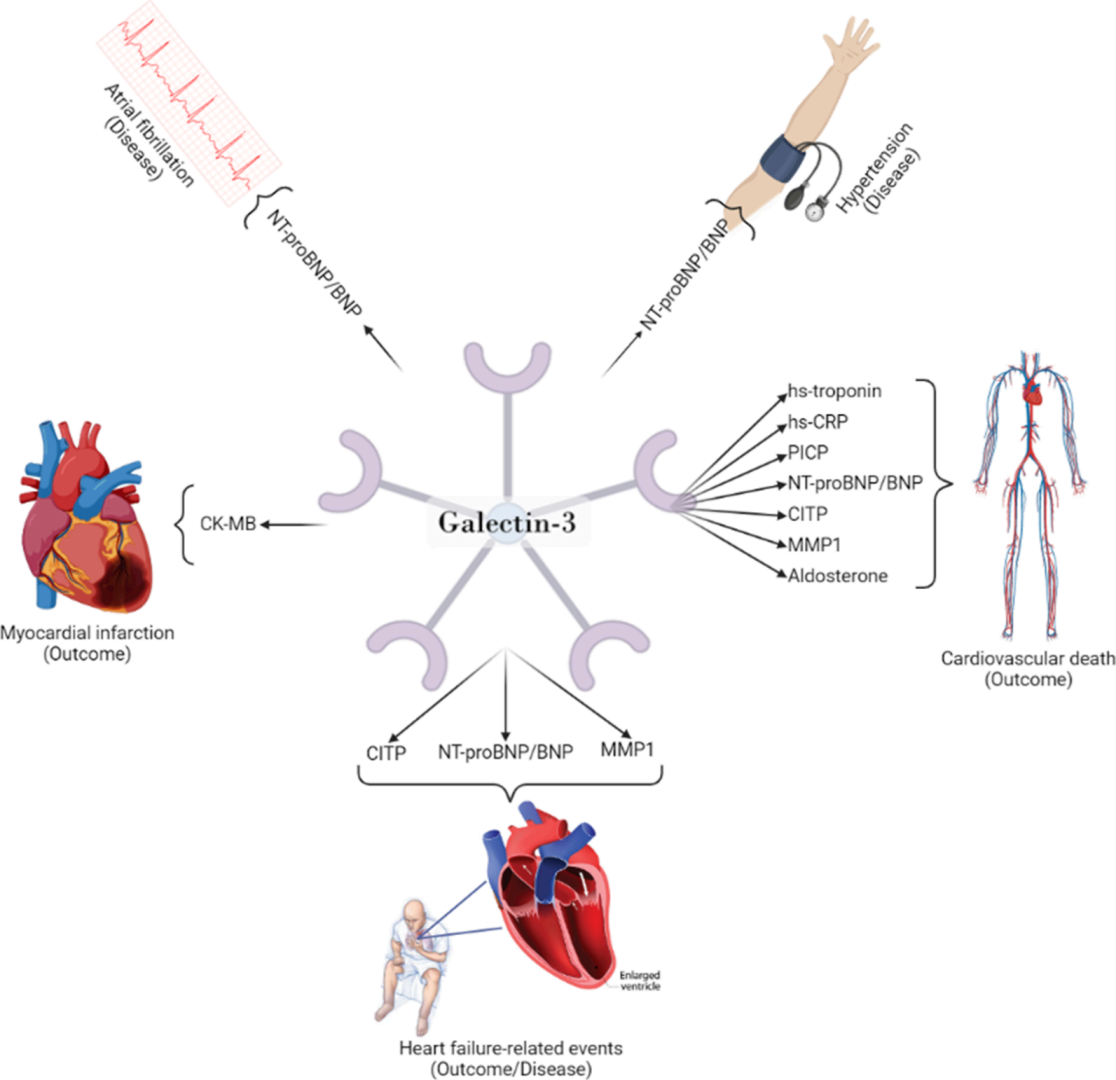
Biomarkers that, combined with Gal-3, have been associated with several cardiovascular diseases and outcomes, specifically with risk assessment, disease prognosis, and diagnosis.

### Potential of Gal-3 as an Outcome Predictor

Much evidence shows the potential of Gal-3 for outcome prediction in the setting of atherosclerosis-rooted CVDs. For instance when assessed immediately after MI, Gal-3 could predict left ventricular ejection fraction and infarct size after 4 months, further supporting the role of this protein in cardiac remodeling after acute MI.^49^ Serum Gal-3 levels were also linked with atrial fibrillation, specifically with the left atrial diameter in patients with paroxysmal atrial fibrillation.^40^ Patients with atrial fibrillation have a significantly increased risk of stroke, even at a subclinical level of atrial fibrillation.^73^ The correlation between Gal-3 levels and LAVI may be of particular interest, since LAVI is a strong predictor of the risk of stroke and mortality.^34,35^ This was associated with a composite outcome of death and vascular events, as well as with stroke recurrence.^36^ Moreover, the combination of elevated Gal-3 and low high-density lipoprotein cholesterol (HDL-C) was associated with primary outcome (stroke onset), recurrent stroke, and vascular events within 1 year after an ischemic stroke. This combination was then suggested to be of clinical use to identify individuals at risk of poor prognosis after ischemic stroke.^43^ Additionally, Gal-3 seems to be independently associated with an increased risk of major disability or death after a stroke event, indicating that this protein may have potential as a prognostic biomarker in ischemic stroke.^38^

The inability of the heart to pump blood across the body efficiently is known as HF and is often the consequence of many CVDs, some of which were discussed previously in this reanalysis.^74^ The complexity of this disease increases the difficulty in finding a clinically viable biomarker panel.^75^ Nonetheless, some clinical trials have focused on this specific condition by using Gal-3 as a monitoring and diagnosing tool. A clinical study led by Tymińska et al. observed that Gal-3 levels were higher in patients at one year follow-up after HF onset when compared to patients who did not reach that primary end point.^33^ Higher Gal-3 was independently associated with worse outcomes, both fatal and nonfatal, in HF.^54^ Elevated levels of Gal-3 have also been correlated with cardiovascular death,^29^ hospitalization by HF, worsening of HF,^31^ and acute HF events.^61^ Overall, Gal-3 has been shown to be a predictor of HF by univariate and multivariate logistic regression analyses.^33^

Gal-3 levels were also able to identify HF patients with a low risk of mortality and of HF rehospitalizations after an acute HF event (30–180 days after).^32^

### Gal-3 and Impact of Different Therapies on CVDs

Spironolactone, an aldosterone (mineralocorticoid) receptor antagonist, is a drug commonly used to treat HF by promoting diuresis and reducing blood pressure, thus reducing myocardial fibrosis and improving left ventricular function.^51,76^ Spironolactone has been shown to reduce total and cardiovascular mortality in HF with reduced ejection fraction (HFrEF).^51^ In clinical trials that assessed the efficacy of this drug through Gal-3 monitoring, it was observed that serum Gal-3 was not associated with reductions in circulating collagen and fibrosis biomarkers in response to spironolactone, including procollagen type III N-terminal propeptide (P3NP), collagen type I C-terminal telopeptide (CITP), and procollagen type I carboxy-terminal propeptide (PICP), which reflect extracellular matrix turnover and myocardial fibrosis.^41,51,52^ In a longitudinal study (0, 6, and 12 months), Gal-3 levels were found elevated in patients with stable HF with preserved ejection fraction (HFpEF). Although spironolactone was not correlated with Gal-3 levels, higher levels of galectin-3 were associated with a worse outcome, independent of treatment.^50^ In some trials testing a treatment with canrenone, another mineralocorticoid receptor antagonist of the spironolactone group, it was found that while the effect of canrenone on clinical outcomes remained unaffected by baseline concentrations of fibrosis or cardiac stress biomarkers, the benefits of this drug were greater in patients with higher levels of Gal-3.^54^ This behavior was also observed in acute HF (AHF) patients treated with spironolactone, where those with elevated levels of Gal-3 benefited more from the treatment, showing higher event-free survival.^44^ It was found that initiation and continuation of spironolactone therapy during AHF hospitalization was associated with a lower risk fomr 30 day mortality and rehospitalization.^44^ Furthermore, the association between spironolactone and improved event-free survival was largely driven by its effect in high-risk patients who had elevated levels of prognostic biomarkers such as creatinine, NT-proBNP, sST-2, and Gal-3 and the presence of clinical risk factors such as hypertension and diabetes.^44^

Anyway, Gandhi et al., 2015 found that Gal-3 levels higher than 20 ng/mL were associated with an increased risk for cardiovascular events, independent of the mineralocorticoid treatment.^53^

Gal-3 may also be used to monitor therapies with valsartan. This angiotensin receptor blocker is often used in combination with sacubitril, a neprilysin inhibitor, to reduce the propensity for fibrosis in patients with HF, and they have already shown to contribute to outcome improvement.^42^ Sacubitril/valsartan significantly decrease profibrotic signaling, as measured by fibrosis biomarkers that are altered in HF with reduced ejection, indicating the prognostic potential of these biomarkers, where Gal-3 is included.^42^ Valsartan use was associated with reduced hospitalizations by HF in patients with low Gal-3 (<16.2 ng/mL) but not in HF patients with high Gal-3.^57^

Another group of drugs used to combat cardiovascular diseases is composed of HMG-CoA reductase inhibitors, also known as statins, such as rosuvastatin and simvastatin. These drugs act by decreasing the amount of circulating cholesterol, halting the buildup in the arterial walls, and thus preventing ischemic and thrombotic events.^77,78^ In this regard, Gal-3 allowed to subset the HF patients that could benefit from rosuvastatin therapy (Gal-3 < 19.0 ng/mL).^55,64^ Rosuvastatin was also used in the setting of MI, reducing endocan (a proteoglycan indicator of inflammation in CVDs) as well as Gal-3 levels in patients with this disease.^45,79^ In patients with chronic HF, after a 6 month treatment with simvastatin, interventricular septal thickness, left ventricular end-diastolic diameter, and serum levels of Gal-3 were decreased, suggesting that Gal-3 can be useful to track cardiac function.^56^

Other therapies, such as intravenous furosemide, CD34+ cell therapy, *N*-acetylcysteine (NAC), and eplerenone were occasionally used in clinical trials aiming to treat HF, MI, or cardiac fibrosis.^46,58–60,80^ Gal-3 was found to be associated with a positive response to the treatment in these trials (except furosemide), making this protein a prime target as a clinical biomarker to assess pharmacotherapy effectiveness in reducing the burden of CVDs, especially HF.

## Conclusions

Galectin-3 is an emerging biomarker of cardiovascular disease. The involvement of this protein in fibrosis and cardiac remodeling, among others, has been established, and its diagnostic and prognostic value has been strengthened by multiple clinical trials. An important step in further validating the clinical potential of Gal-3 is through clinical trials. There are some clinical trials regarding cardiovascular diseases with published results that approach and/or use Gal-3 to monitor, prognose and diagnose the said diseases (Table 1). However, these clinical trials present several limitations that can be mitigated in future studies to make strong advancements in incorporating Gal-3 into the clinical setting. Taking into consideration the limitations observed, future clinical trials should consider having an elevated number of patients in the cohort with detailed clinical history and multiple time-point measurements, accompanied by medical exams such as echocardiography, MRI, and computerized axial tomography (CAT)-angiograms. Moreover, one common point across many clinical trials assessed in this reanalysis was the lack of statistical significance of the results when adjusted to multiple factors (i.e., age, sex, cholesterol, diabetes, and smoking status). Therefore, future clinical trials should have better suited inclusion/exclusion criteria for the patients.

Gal-3 seems to have strong associations with several biomarkers, including NT-proBNP—a biomarker for the diagnosis of HF—and LVEF—a strong diagnostic indicator for HF, which promote its potential as a biomarker itself for HF. Moreover, Gal-3 shows associations with other outcomes, cardiovascular diseases, therapies, and comorbidities that further enhance its value as a biomarker tool. However, further studies are required to validate this protein as a biomarker.

## Limitations

The analyzed clinical trials present high variability on cohort size^33,33,41–43,46,52,56,58,61,64^ exclusion and inclusion criteria^36,42,55,64^ and protocol, including analyses not properly adjusted to NT-proBNP or to PICP, which may reflect conditions other than cardiac or vascular fibrosis, such as bone turnover.^29^ Additional methodological heterogeneity arises from missing or incomplete tests and exams, namely, the absence of echocardiogram,^33^ echocardiography,^63,80^ noninvasive imaging tests to quantify the degree of fibrosis,^58^ or cardiac MRI,^80^ which limits direct structural and functional cardiac assessment. Moreover, the studies presented divergent primary aims and end points, further complicating cross-study comparability.

Furthermore, common limitations included the lack or limited measurement of relevant biomarkers, such as parathyroid hormone (PTH);^29^ Gal-3 and sST2 measured only at the control visit and NT-proBNP during hospital stay;^33^ Gal-3 assessed at a limited number of time points;^36,45,48,57,63^ incomplete evaluation of the MMP/TIMP balance;^31^ absence or limited measurement of NT-proBNP;^63,80^ IL-33 not measured;^80^ and renal biomarkers such as creatinine and cystatin C.^63^ Additionally, some of the analyzed clinical trials were retrospective, which may have precluded the identification of potential prognostic markers^51^ and limited the ability to account for unmeasured confounders.^44,57^ Finally, as subgroup analyses of larger clinical trials, some of the studies herein analyzed were hypothesis-generating in nature,^32,44,48,53^ restricting the strength of causal inference.

## Supplementary Material



## Notes

Table S1: Complete data retrieved from clinical trials assessed in this review (PDF)

## Abbreviations

AGTR1: Angiotensin II receptor type 1
AHF: Acute heart failure
BNP: Brain natriuretic peptide 32
CAT: Computerized axial tomography
CABG: Coronary artery bypass graft
CAD: Coronary artery disease
CITP: Collagen type-1 C-terminal telopeptide
CK-MB: Creatine kinase MB
CMIA: Chemiluminescent microparticle immunoassay (CMIA)
COPD: Chronic obstructive pulmonary disease
CRP: C-reactive protein
CV: Cardiovascular
CVDs: Cardiovascular diseases
DPB: Diastolic blood pressure
ECG: Electrocardiogram
eGFR: estimated glomerular filtration rate
ELISA: Enzyme-linked immunosorbent assay
E’: Early diastolic mitral annular velocity
E/E’: Early diastolic transmitral velocity to early diastolic mitral annular velocity
Gal-3: Galectin-3
GDF-15: Growth differentiation factor 15
GFAP: Glial fibrillary acidic protein
HDL-C: Low high-density lipoprotein cholesterol
HF: Heart failure
HFpEF: Heart failure with preserved ejection fraction
HFrEF: Heart failure with reduced ejection fraction
HMGCR/HMG-CoA: 3-Hydroxy-3methylglutaryl-coA reductase
hs-CRP: High-sensitivity C-reactive protein
hs-cTnI: High sensitivity cardiac troponin I
ICAM 1: Intercellular adhesion molecule 1
IL: Interleukin
LAVI: Left atrial volume index
LVDD: Left ventricular diastolic dysfunction
LVEF: Left ventricle ejection fraction
LVMI: Left ventricular myocardial index
LVSD: Left ventricular systolic disfunction
MACE: Major acute cardiovascular events
MI: Myocardial infarction
MMP: Matrix metalloproteinases
MPO: Myeloperoxidase
MRA: Mineralocorticoid receptor antagonists
MRI: Magnetic resonance imaging
MTWA: ECG-based microvolt T-wave alternans
NAC: N-acetylcysteine
NR3C2: Nuclear receptor subfamily 3 group C member 2
NT-proBNP: NT-Pro-B-type matriuretic protein
P3NP: Procollagen III N-terminal propeptide
PCI: Percutaneous coronary intervention
PICP: Procollagen type I carboxy-terminal propeptide
PTH: Parathyroid hormone
REN: Renin
SBP: Systolic blood pressure
sST2: Soluble interleukin 1 receptor-like 1
TIMP1: Metallopeptidase inhibitor 1
TNF-α: Tumor necrosis factor
VCAM 1: Vascular cell adhesion protein 1
vWF: van Willebrand factor
