# Supplementary material for: Exploring Galectin‑3 as an Emerging Frontier in Biomarker Research for Clinical Trials Approaching Cardiovascular Diseases
Source: ACS Pharmacol Transl Sci. 2026 Mar 11;9(4):771–83. doi: 10.1021/acsptsci.5c00671 (PMC13077495; doi:10.1021/acsptsci.5c00671)
Supplement: Supplementary file 1 [file pt5c00671_si_001.pdf]

Exploring Galectin-3 as an Emerging Frontier in Biomarker Research for Clinical Trials Approaching Cardiovascular Diseases

Luís Perpétuo[1,2,*]

[1]iBiMED, Department of Medical Sciences, University of Aveiro, Campus Universitário de Santiago, 3810-193 Aveiro, Portugal
[2]Cardiovascular R&D Centre – RISE-Health, Department of Surgery and Physiology, Faculty of Medicine of the University of Porto, Alameda Professor Hernâni Monteiro 4200-319 Porto, Portugal
ORCID 0000-0002-8207-7700

Addressing author: luisperpetuo@ua.pt

This document presents the supplementary information relevant to the review article "Exploring Galectin-3 as an Emerging Frontier in Biomarker Research for Clinical Trials Approaching Cardiovascular Diseases".

Supplementary Table 1 – Complete data retrieved from clinical trials assessed in this review

	MI	HF	HF	HF	HF	HF
Paper reference	47	48	49	56	58	61
N of associations with gal-3	7	9	10	9	9	6
N	1069 (total=2970)	205 (total=413)	488 (total=1462)	69 (total=208)	728 (total=1462)	207 (total=415)
Galectin-3 (ng/mL)	> 8.65	13.3 [12.0-15.8]	>21.6	21.0 [18.1-24.7]	>19.0	>12.1
Age (years)	64.2 ± 10.89*	65.0 ± 8.3*	74 ± 7*	71 [65-79]*	73 ± 7*	68 ± 8*
Females (%)	45.89*	16.6	29*	46	27*	-
BMI (kg/m2)	24.81 ± 3.18*	27.0 ± 3.8	27.3 ± 5	32.8 [28.1-39.6]	27.2 ± 4.98	29.3 ± 3.6
SBP (mmHg)	166.7 ± 17.19	128.8 ± 17.9	129 ± 17*	-	129 ± 17*	133.8 ± 18.8
BDP (mmHg)	96.26 ± 11.37	78.2 ± 8.5	76 ± 10*	-	79 ± 9*	77.5 ± 11.6
HR (beats/min)	-	67.9 ± 11.5	72 ± 11*	-	72 ± 11*	-
Current smoker (%)	29.81*	-	11	-	12	-
Hypertension (%)	80.88*	49.3	68	91	69	94.7*
Diabetes (%)	26.1*	26.3*	28	57*	28*	19.8
Myocardial infarction (%)	-	45.9	67	-	65*	17.9
CABG or PCI (%)	-	-	25*	-	23*	8.7
Ischemic etiology (%)	-	49.3	-	-	-	-
Diagnosis of HF > 12 months (%)	-	72.7	-	-	-	-
Prior hospitalization for HF (%)	-	56.1*	-	45	-	33.8
Prior stroke (%)	-	3.4	12	-	13	-

COPD (%)	-	14.2*	-	19	-	3.9
Peripheral vascular disease (%)	-	5.4*	-	-	-	5.8*
LVEF (%)	-	38.5 ± 8.6*	30.9 ± 6.8*	-	31 ± 7*	67.8 ± 7.9
LVEDV (mL)	-	165.4 ± 68.1	-	-	-	-
LV mass (g)	-	273 ± 80	-	-	-	-
RWT	-	0.33 ± 0.06	-	-	-	-
Left atrial diameter (cm)	-	4.3 ± 0.8*	-	-	-	-
E/A	-	1.33 ± 1.11	-	-	-	-
Aspirin (%)	34.72	-	-	-	-	18.8*
Diuretics (%)	-	78.1*	92*	93*	89*	63.8*
B-blockers (%)	54.26*	78.5	73	80	74	79.2*
ACEi or ARBs (%)		100*	81 / 43*	75	81	81.2
Digitalis (%)	-	25.4	32*	-	31	-
Nitrates (%)	-	30.2	-	-	-	-
Statins (%)	2.84	40.5	-	-	-	58.5
Antiplatelets (%)	96.38	54.6	-	-	-	54.6
Serum creatinine (mg/dL)	-	1.11 ± 0.28*	-	1.3 [1.0-1.7]*	-	-
Serum potassium (mEq/L)	-	4.4 ± 0.4	-	-	-	-
WBC (mm-3)	-	6,769 ± 1,666	-	-	-	-
PIIINP (ug/L)	-	5.8 [4.4-7.4]*	-	8.3 [6.7-11.5]*	-	-
Aldosterone (pg/mL)	-	125 [75-187]	-	-	-	-
NT-proBNP (pg/mL)	-	-	2089 [820-3983]*	1187 [397-2205]*	213.2 [88.7-444.2]*	192 [93-377]*
BNP (ng/L)	-	112 [52-222]*	-	-	-	-
hsCRP (mg/L)	-	-	4.7 [2.2-9.8]*	3.8 [1.7-8.5]	4.6 [2.1-8.9]*	-
hs-TnTa (ng/mL)	-	-	-	10.2 [6.7-20.9]	-	-
Total cholesterol (mmol/L)	5.3 ± 1.3*	-	5.24 ± 1.17	-	5.23 ± 1.13	-
LDL cholesterol (mmol/L)	3.07 ± 1.05*	-	3.62 ± 1.02	-	3.63 ± 1.04	-
HDL cholesterol (mmol/L)	-	-	1.22 ± 0.35	-	1.22 ± 0.35	-
Triglycerides (mmol/L)	1.89 ± 4.53*	-	2.08 ± 1.51	-	2.07 ± 1.41	-
eGFR (mL/min per 1.73 m2)	-	-	49 ± 13*	51 [35-68]*	52.0 ± 13.3*	72.5 ± 18.2*
ApoB/ApoA-1 ratio)	-	-	0.91 ± 0.27*	-	0.9 ± 0.26	-
Albumin (g/L)	-	-	-	-	-	-
Sodium (mmol/L)	-	-	-	-	-	140.4 ± 2.7
Potassium (mmol/L)	-	-	-	-	-	4.2 ± 0.4
Uric acid (mg/dL)	-	-	-	8.0 [6.4-9.6]*	-	-
